# Supplementary material for: Giant impacts stochastically change the internal pressures of terrestrial planets
Source: Sci Adv. 2019 Sep 4;5(9):eaav3746. doi: 10.1126/sciadv.aav3746 (PMC6726449; doi:10.1126/sciadv.aav3746)
Supplement: http://advances.sciencemag.org/cgi/content/full/5/9/eaav3746/DC1 [file supp_5_9_eaav3746__index.html]

Science Advances | Science AdvancesAAASSearchScience AdvancesMenu

## Supplementary Materials

**The PDF file includes:**

- Fig. S1. Pressure change in cooling from the CoRoL to a magma ocean planet.
- Fig. S2. Effect of forming the Moon on internal pressures.
- Fig. S3. Isentropes for the M-ANEOS–derived forsterite equation of state in pressure-temperature space.
- Fig. S4. Effect of thermal state on the pressure in condensed bodies.
- Fig. S5. Sensitivity to the number of concentric layers used in HERCULES.
- Fig. S6. Sensitivity to the number of points used to describe each surface in HERCULES.
- Fig. S7. Sensitivity to the maximum spherical harmonic degree used in HERCULES.
- Fig. S8. Comparison of pressures calculated using SPH and HERCULES.
- Legend for table S1

Download PDF

**Other Supplementary Material for this manuscript includes the following:**

- Table S1 (.csv format). Impact parameters and properties of resulting bodies at different stages in evolution.

**Files in this Data Supplement:**

- Adobe PDF - aav3746\_SM.pdf
