# Supplementary material for: Giant impacts stochastically change the internal pressures of terrestrial planets
Source: Sci Adv. 2019 Sep 4;5(9):eaav3746. doi: 10.1126/sciadv.aav3746 (PMC6726449; doi:10.1126/sciadv.aav3746)
Supplement: Download PDF [file aav3746_SM.pdf]

Science Advances
AAAS

advances.sciencemag.org/cgi/content/full/5/9/eaav3746/DC1

Supplementary Materials for

Giant impacts stochastically change the internal pressures of terrestrial planets

Simon J. Lock* and Sarah T. Stewart

*Corresponding author. Email: slock@caltech.edu

Published 4 September 2019, *Sci. Adv.* **5**, eaav3746 (2019)
DOI: 10.1126/sciadv.aav3746

The PDF file includes:

Fig. S1. Pressure change in cooling from the CoRoL to a magma ocean planet.
Fig. S2. Effect of forming the Moon on internal pressures.
Fig. S3. Isentropes for the M-ANEOS–derived forsterite equation of state in pressure-temperature space.
Fig. S4. Effect of thermal state on the pressure in condensed bodies.
Fig. S5. Sensitivity to the number of concentric layers used in HERCULES.
Fig. S6. Sensitivity to the number of points used to describe each surface in HERCULES.
Fig. S7. Sensitivity to the maximum spherical harmonic degree used in HERCULES.
Fig. S8. Comparison of pressures calculated using SPH and HERCULES.
Legend for table S1

Other Supplementary Material for this manuscript includes the following:

(available at advances.sciencemag.org/cgi/content/full/5/9/eaav3746/DC1)

Table S1 (.csv format). Impact parameters and properties of resulting bodies at different stages in evolution.

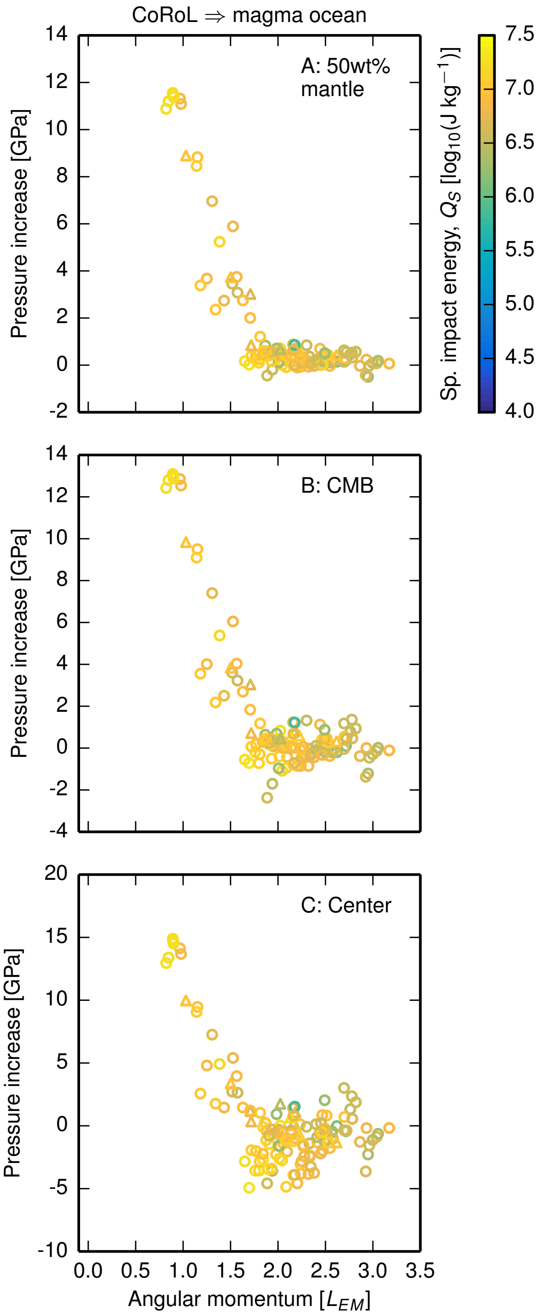

Fig. S1. Pressure change in cooling from the CoRoL to a magma ocean planet. The pressure change in cooling from the corotation limit to a magma ocean is small for high-angular-momentum bodies. Panels show the pressure difference between a body just below the CoRoL and a magma-ocean planet of the same angular momentum at the middle of the mantle by mass (A), the core-mantle boundary (B) and at the center (C) of the body. Colors and symbols are the same as in Figure 2. To calculate the pressures in bodies at the corotation limit, we used a thermal profile that approximates the thermally-stratified, partially-vaporized structure of post-impact bodies during cooling (see Materials and Methods). The competing effects of changes in moment of inertia and bulk density during condensation means that some bodies have slightly higher internal pressures when they are just below the the CoRoL than when a magma-ocean planet.

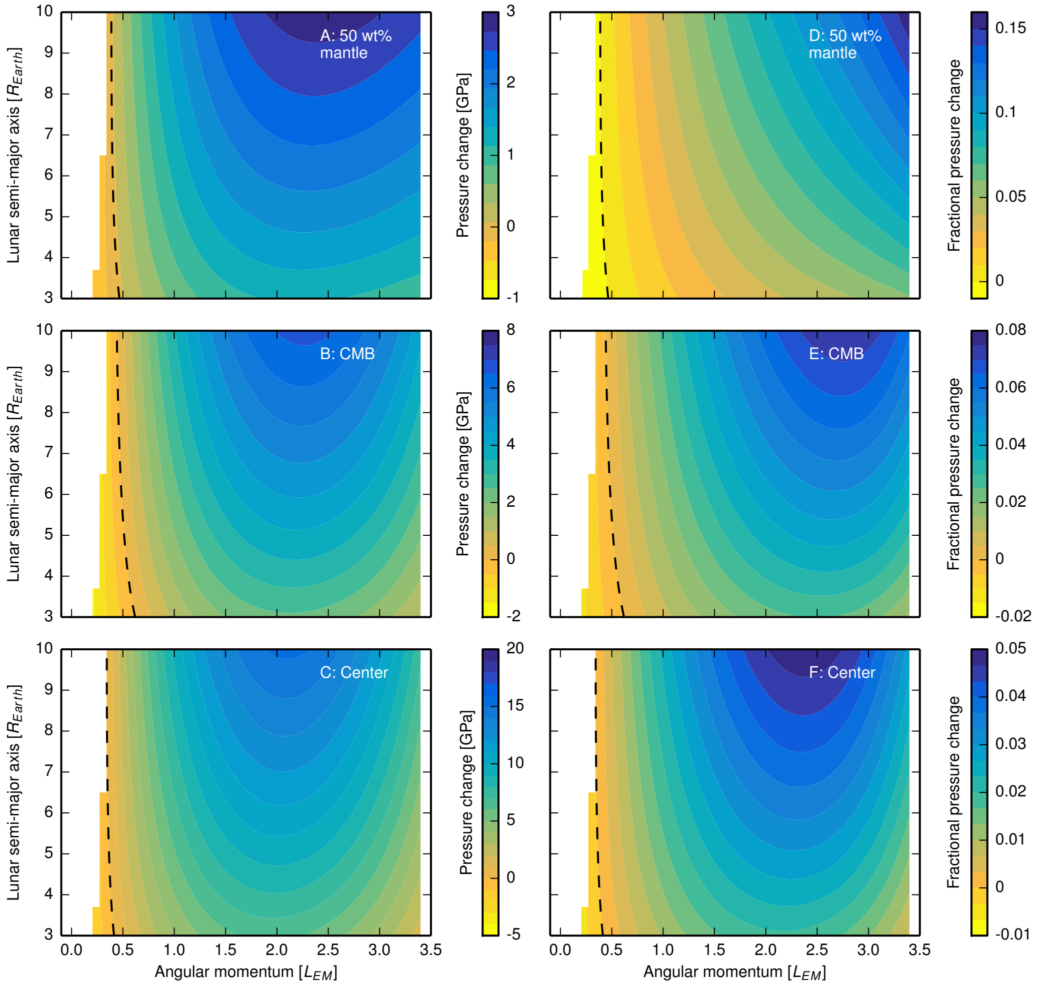

Fig. S2. Effect of forming the Moon on internal pressures. The formation of a close-in moon has only a minor effect on the internal pressures in a magma-ocean after a giant impact. Shown is the absolute (left column) and fractional (right column) difference in the pressures in an Earth-like magma-ocean planet orbited by a tidally-locked Moon at a given semi-major axis (y-axis) and a system with the same total angular momentum, mass, and composition, but with all the mass combined into a single magma-ocean planet. Panels show the pressure difference at the middle of the mantle by mass (A, D), the core-mantle boundary (B, E) and at the center (C, F) of the body. The dashed lines indicate the locus of points for which there is no difference in pressure between the two systems.

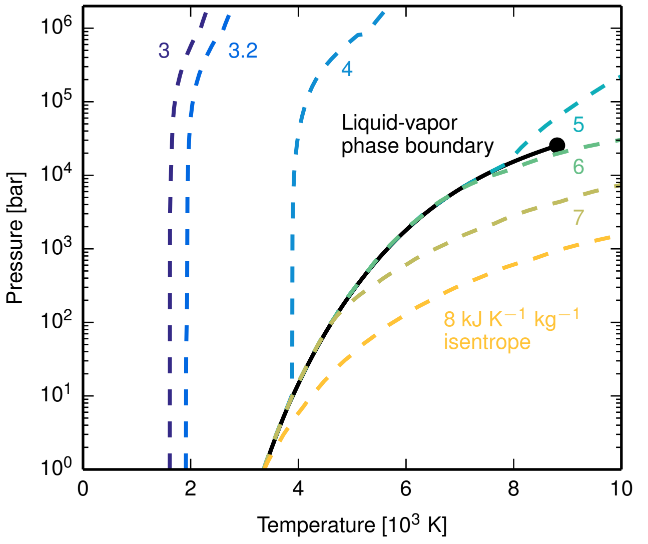

Fig. S3. Isentropes for the M-ANEOS–derived forsterite equation of state in pressure-temperature space. Each colored line is an isentrope for the specific entropy given by the number of the same color in kJ K^{-1} kg^{-1}. The black line is the liquid-vapor phase boundary. The black dot is the critical point. Adapted from (2).

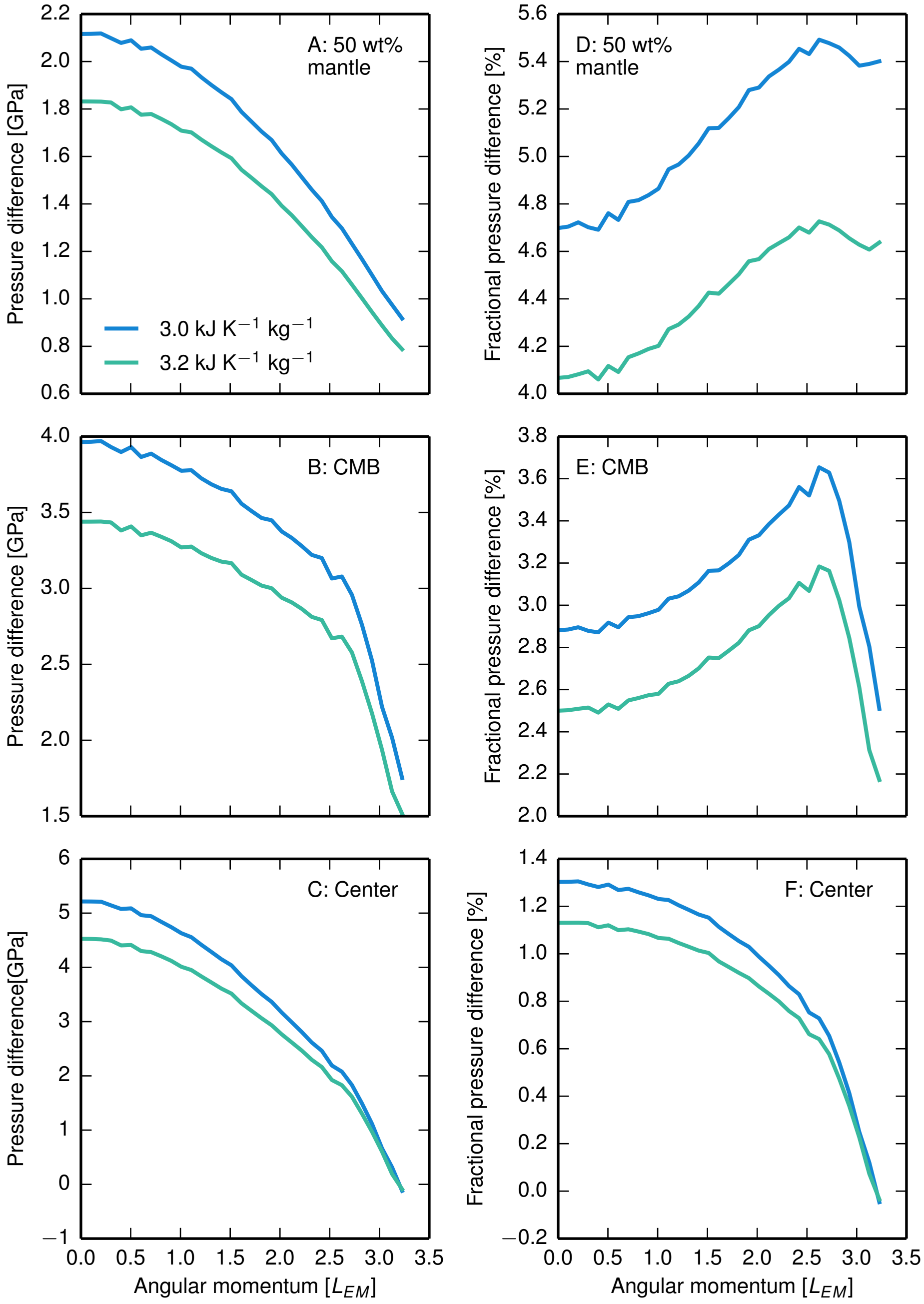

Fig. S4. Effect of thermal state on the pressure in condensed bodies. The thermal state of condensed terrestrial bodies makes only a small difference to the internal pressure. The absolute (left column) and fractional (right column) difference in pressure between bodies that have a mantle entropy of 4 kJ K^{-1} kg^{-1} (the thermal state used for magma-ocean planets elsewhere in this paper) and bodies with mantle entropies of 3 and 3.2 kJ K^{-1} kg^{-1} are shown by the blue and green lines respectively. Mantle specific entropies of 3 and 3.2 kJ K^{-1} kg^{-1} correspond to mantle potential temperatures of ~ 1600 K and ~ 1900 K, similar to the present-day and early terrestrial mantle respectively. Rows show the pressure difference in the mid mantle (A, D), core-mantle boundary (B, E), and center of the body (C, F).

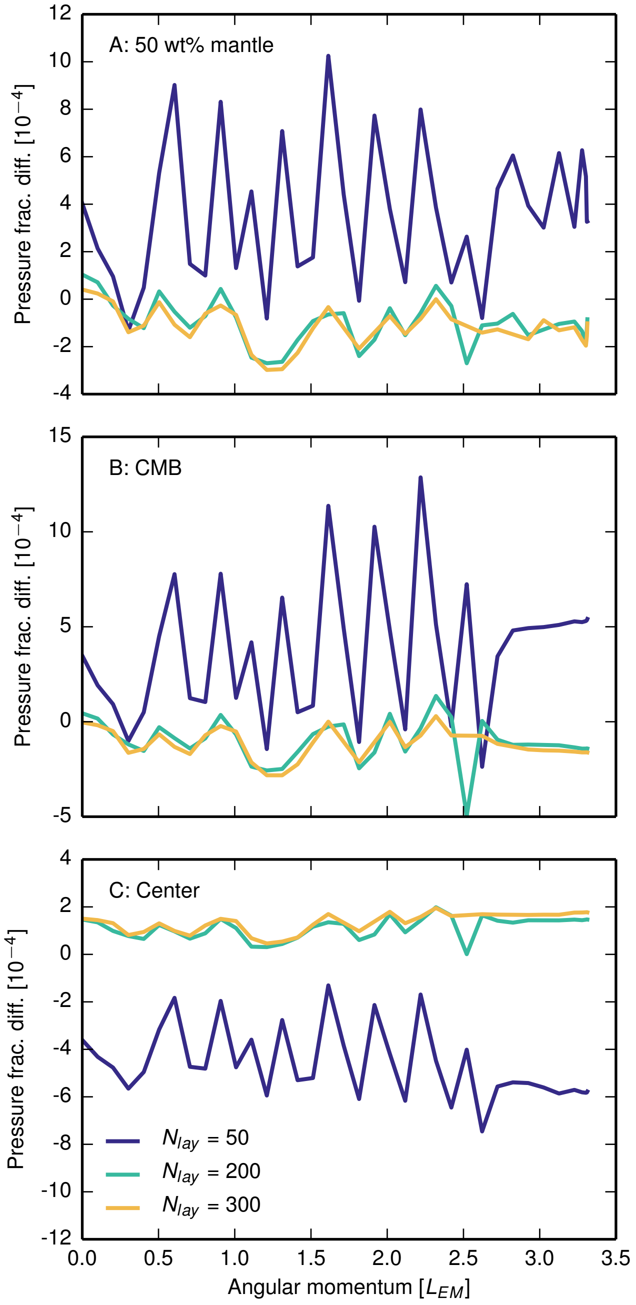

Fig. S5. Sensitivity to the number of concentric layers used in HERCULES. The internal pressures calculated using HERCULES are only weakly dependent on the number of concentric layers used (N_{lay}). Shown are the fractional difference in pressure in bodies with a range of angular momenta calculated using HERCULES with a given number of concentric layers (N_{lay}, colored lines) and the same body calculated with N_{lay} = 100 as used elsewhere in this paper. Panels show the difference in the pressure at the middle of the mantle by mass (A), the core-mantle boundary (B) and the center (C) of the bodies.

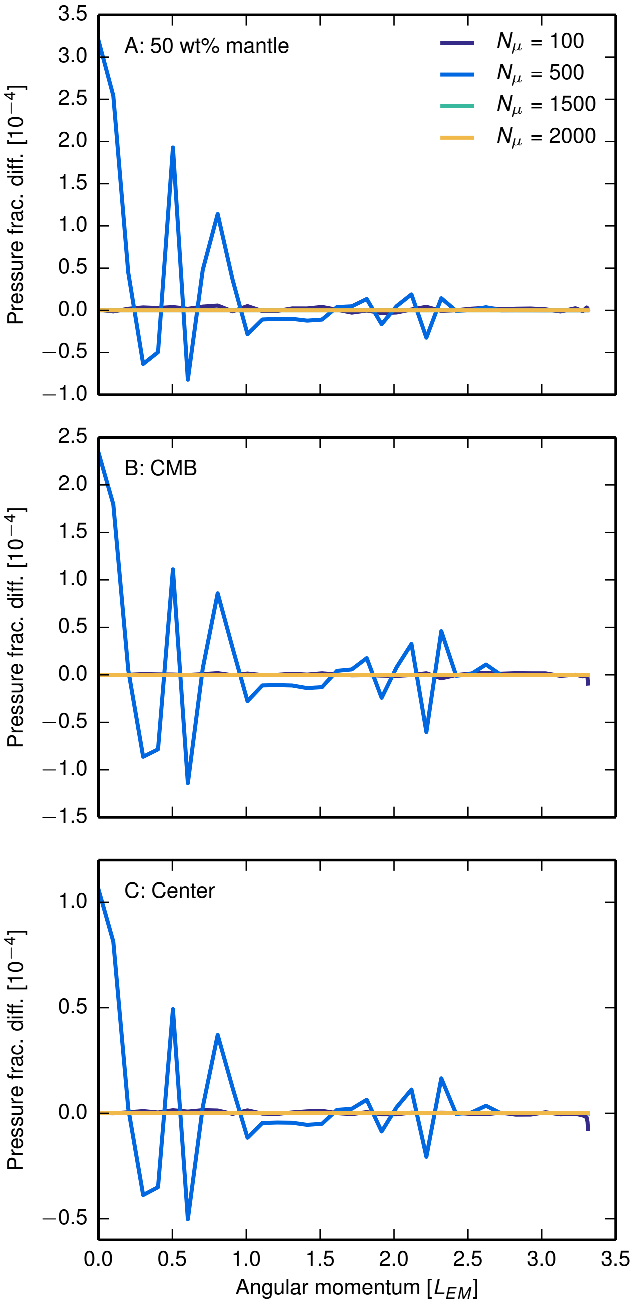

Fig. S6. Sensitivity to the number of points used to describe each surface in HERCULES. The internal pressures calculated in HERCULES are only weakly dependent on the number of points used to describe each equipotential surface (N_μ). Shown are the fractional differences in internal pressures between bodies with a range of angular momenta calculated using HERCULES with varying N_μ (colored lines) compared to the same body calculated using the parameters used elsewhere in this paper (N_μ = 1000). Panels show the difference in the pressure at the middle of the mantle by mass (A), the core-mantle boundary (B) and the center (C) of the bodies.

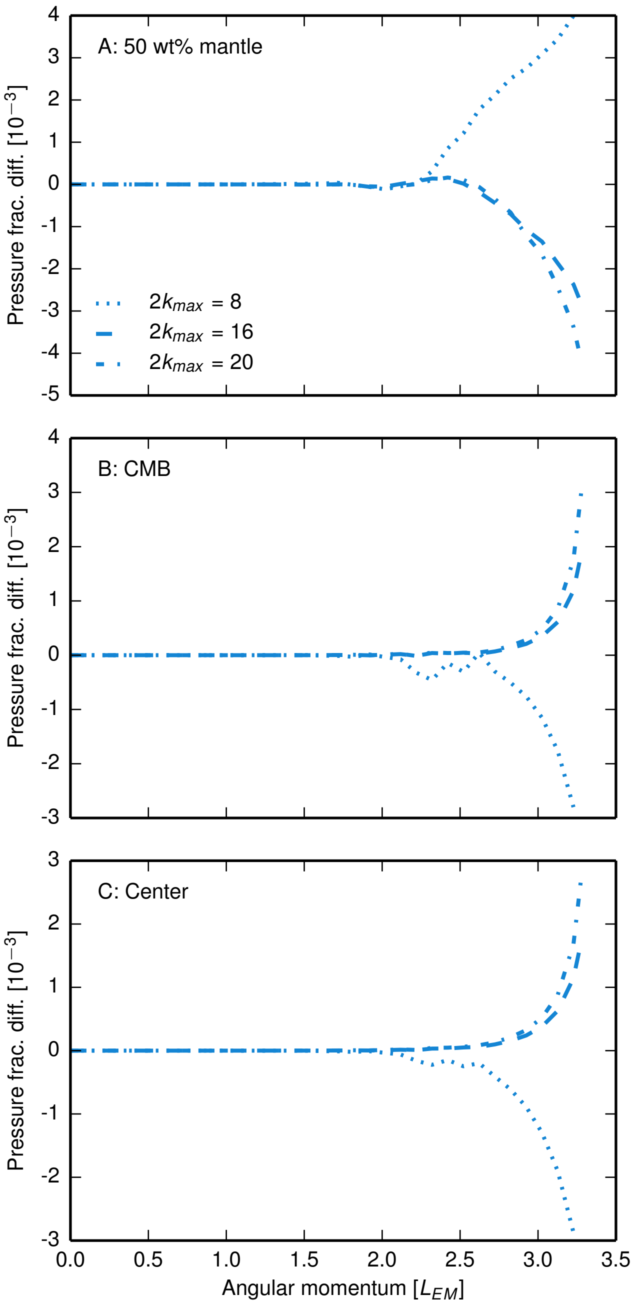

Fig. S7. Sensitivity to the maximum spherical harmonic degree used in HERCULES. The internal pressures calculated in HERCULES are only weakly dependent on the maximum spherical harmonic degree included ($2k_{max}$). Shown are the fractional differences in internal pressures between bodies with a range of angular momenta calculated using HERCULES with different k_{max} (lines) compared to the same body calculated using the parameters used elsewhere in this paper (k_{max} = 6). Panels show the differences in the pressure at the center (A), the core-mantle boundary (B) and the middle of the mantle by mass (C) of each body. Panels show the difference in the pressure at the middle of the mantle by mass (A), the core-mantle boundary (B) and the center (C) of the bodies.

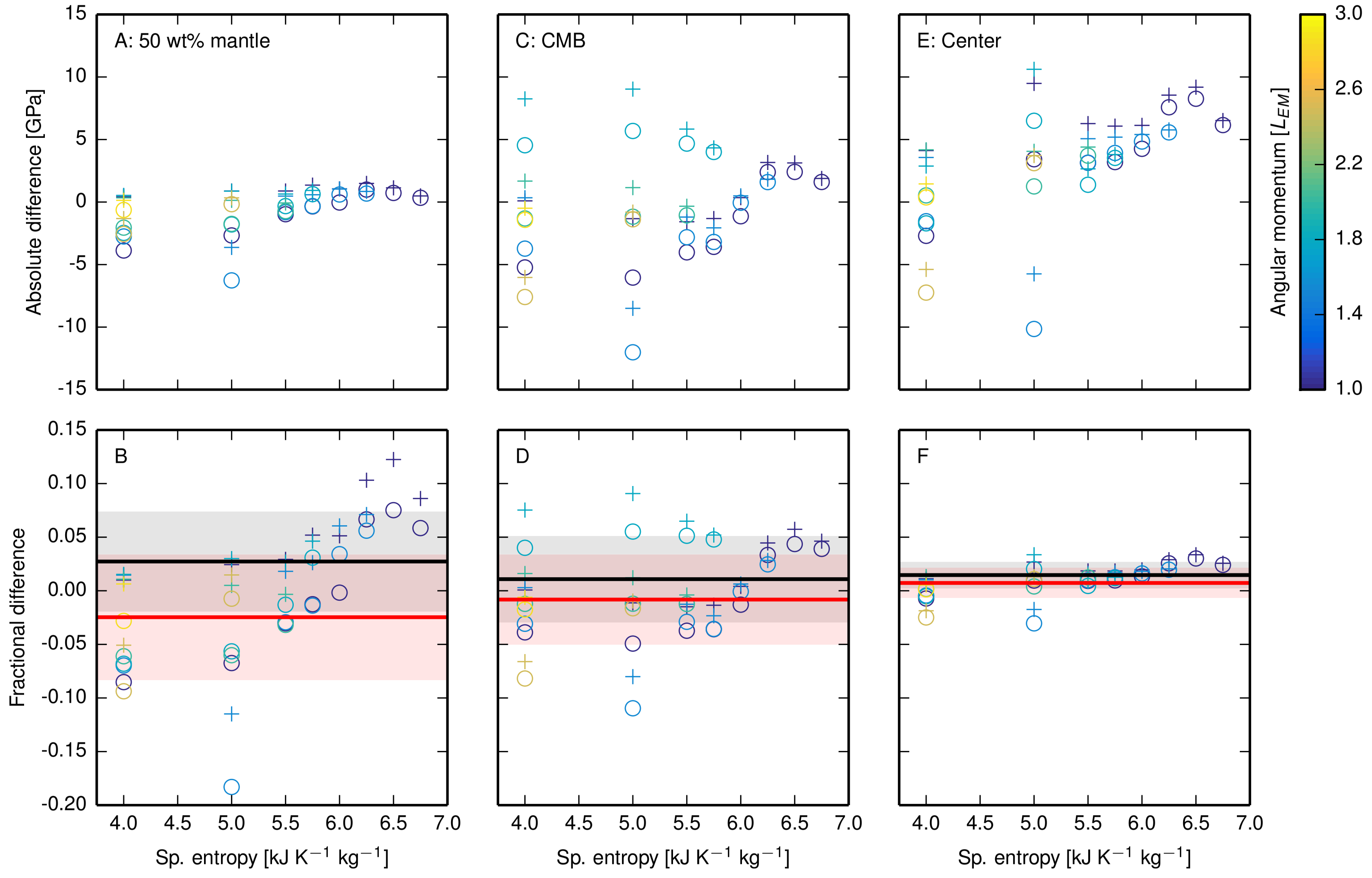

Fig. S8. Comparison of pressures calculated using SPH and HERCULES. Planetary structures calculated using SPH and HERCULES have similar internal pressures. Rows show the absolute (top) and fractional (bottom) difference in pressures between SPH and HERCULES calculations. Columns show the pressure difference in different locations in the Earth-mass bodies with different angular momenta (colors) and isentropic mantles of varying specific entropy (x-axis). Comparisons were made to HERCULES planets with a bounding pressure of both 10 bar (+) and a pressure equivalent to the lowest pressure in the midplane of the SPH structure (○). The solid black and red lines in the bottom row show the mean fractional pressure differences using HERCULES planets with 10 bar and the maximum SPH pressure respectively. The shaded area shows one standard deviation in the fractional errors. The shape and pressure contours for the SPH bodies plotted here are shown in Figure 4 of (2).

Table S1. Impact parameters and properties of resulting bodies at different stages in evolution. Summary of SPH impact simulations used in this paper and properties of their post-impact states. Most calculations were reported in (2) but additional simulations of near mutual escape velocity collisions with high impactor to target mass ratios have also been performed. For each impact, the table includes: an index number; target mass M_{t}; number of SPH particles in target, N_{t}; target equatorial radius, R_{t}; target angular momentum, L_{t}; target 50% mantle pressure, p_{50}^{t}; target core-mantle boundary (CMB) pressure, $p_{\mathrm{CMB}}^{\mathrm{t}}$; target central pressure, $p_{\mathrm{center}}^{\mathrm{t}}$; projectile mass, M_{p}; number of SPH particles in projectile, N_{p}; projectile radius, R_{p}; projectile angular momentum, L_{p}; impact velocity, V_{i}; impact parameter, b; modified specific energy, Q_{S}; final simulation time; bound mass of post-impact structure, M_{bnd}; core mass fraction of post-impact body, f_{core}; bound mass angular momentum, L_{bnd}; angular velocity of the dense ($\rho > 1000$ kg m^{-3}) region of post-impact structure, ω_{ρ}; spin period of dense region, T_{ρ}; moment of inertia of bound mass, I_{bnd}; post-impact 50% mantle pressure, p_{50}^{bnd}; post-impact CMB pressure, $p_{\mathrm{CMB}}^{\mathrm{bnd}}$; post-impact central pressure, $p_{\mathrm{center}}^{\mathrm{bnd}}$; 50% mantle pressure in a body of the same mass, core fraction, and angular momenta but with a hot partially-vaporized, thermally-stratified thermal structure with an upper mantle entropy such that the body is at the CoRoL, p_{50}^{CoRoL}; CMB pressure at the CoRoL, $p_{\mathrm{CMB}}^{\mathrm{CoRoL}}$; central pressure at the CoRoL, $p_{\mathrm{center}}^{\mathrm{CoRoL}}$; angular velocity of a magma-ocean planet with the same mass, angular momentum and core-mass fraction as the post-impact body, ω_{MO}; moment of inertia of the corresponding magma-ocean planet, I_{MO}; 50% mantle pressure in corresponding magma-ocean planet, p_{50}^{MO}; CMB pressure in corresponding magma-ocean planet, $p_{\mathrm{CMB}}^{\mathrm{MO}}$; central pressure in corresponding magma-ocean planet, $p_{\mathrm{center}}^{\mathrm{MO}}$; 50% mantle pressure of a magma-ocean planet with the same mass and core-mass fraction as the post-impact body but with an angular momentum corresponding to that of the Earth's when the lunar orbit reached the Cassini-state transition, $p_{50}^{\mathrm{Cassini}}$; CMB pressure in corresponding magma-ocean planet at the Cassini state transition, $p_{\mathrm{CMB}}^{\mathrm{Cassini}}$; central pressure in corresponding magma-ocean planet at the Cassini-state transition, $p_{\mathrm{center}}^{\mathrm{Cassini}}$; and post-impact structure dynamical class.
